# Supplementary material for: Effective prediction of biosynthetic pathway genes involved in bioactive polyphyllins in Paris polyphylla
Source: Commun Biol. 2022 Jan 13;5:50. doi: 10.1038/s42003-022-03000-z (PMC8758714; doi:10.1038/s42003-022-03000-z)
Supplement: Supplementary file 2 — Supplementary Information [file 42003_2022_3000_MOESM2_ESM.pdf]

Supplementary Information

Effective prediction of biosynthetic pathway genes involved in bioactive polyphyllins in *Paris polyphylla*

Xin Hua[1,&], Wei Song[2, &], Kangzong Wang[1,&], Xue Yin[1,] , Changqi Hao[1] , Baozhong Duan[3], Zhichao Xu[1, 4,] *, Tongbing Su[5,6]*, Zheyong Xue[1]*

[1] Key Laboratory of Saline-alkali Vegetation Ecology Restoration (Northeast Forestry University)，Ministry of Education, Harbin, China

[2] College of Pharmacy, Zhejiang Chinese Medical University, Hangzhou, China

[3] College of Pharmaceutical Science, Dali University, Dali, China

[4] Institute of Medicinal Plant Development, Chinese Academy of Medical Sciences & Peking Union Medical College, Beijing, China

[5] Beijing Vegetable Research Center (BVRC), Beijing Academy of Agriculture and Forestry Science (BAAFS), Beijing, China

[6] National Engineering Research Center for Vegetables, Beijing 100097, China

[&] X.H., W.S. and K.W. contributed equally.

* corresponding author: Zheyong Xue (zyxue@nefu.edu.cn), Tongbing Su (sutongbing@nercv.org) or Zhichao Xu (zcxu@implad.ac.cn).

Supplementary Figures

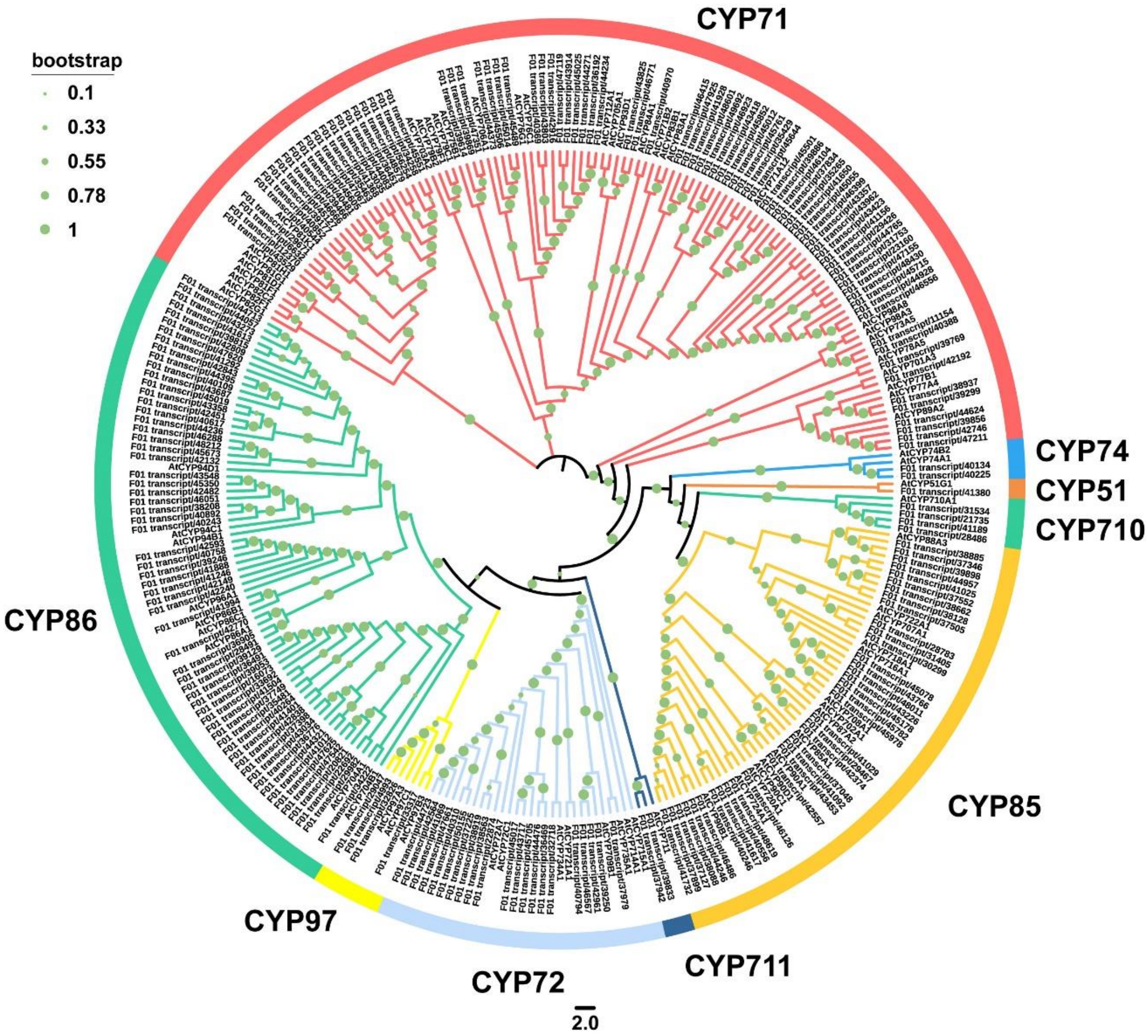

Supplementary Figure 1. Phylogenetic tree of CYPs. Selected Arabidopsis CYPs were included in the analysis to define CYP families. The evolutionary history was inferred using the Neighbor-Joining method. The bootstrap consensus tree inferred from 1000 replicates is taken to represent the evolutionary history of the taxa analyzed. Evolutionary analyses were conducted in MEGA6. According to the reported family CYP genes of *Arabidopsis thaliana*, the phylogenetic tree is divided into CYP51, CYP71, CYP710, CYP711, CYP72, CYP74, CYP85, CYP86, and CYP94.

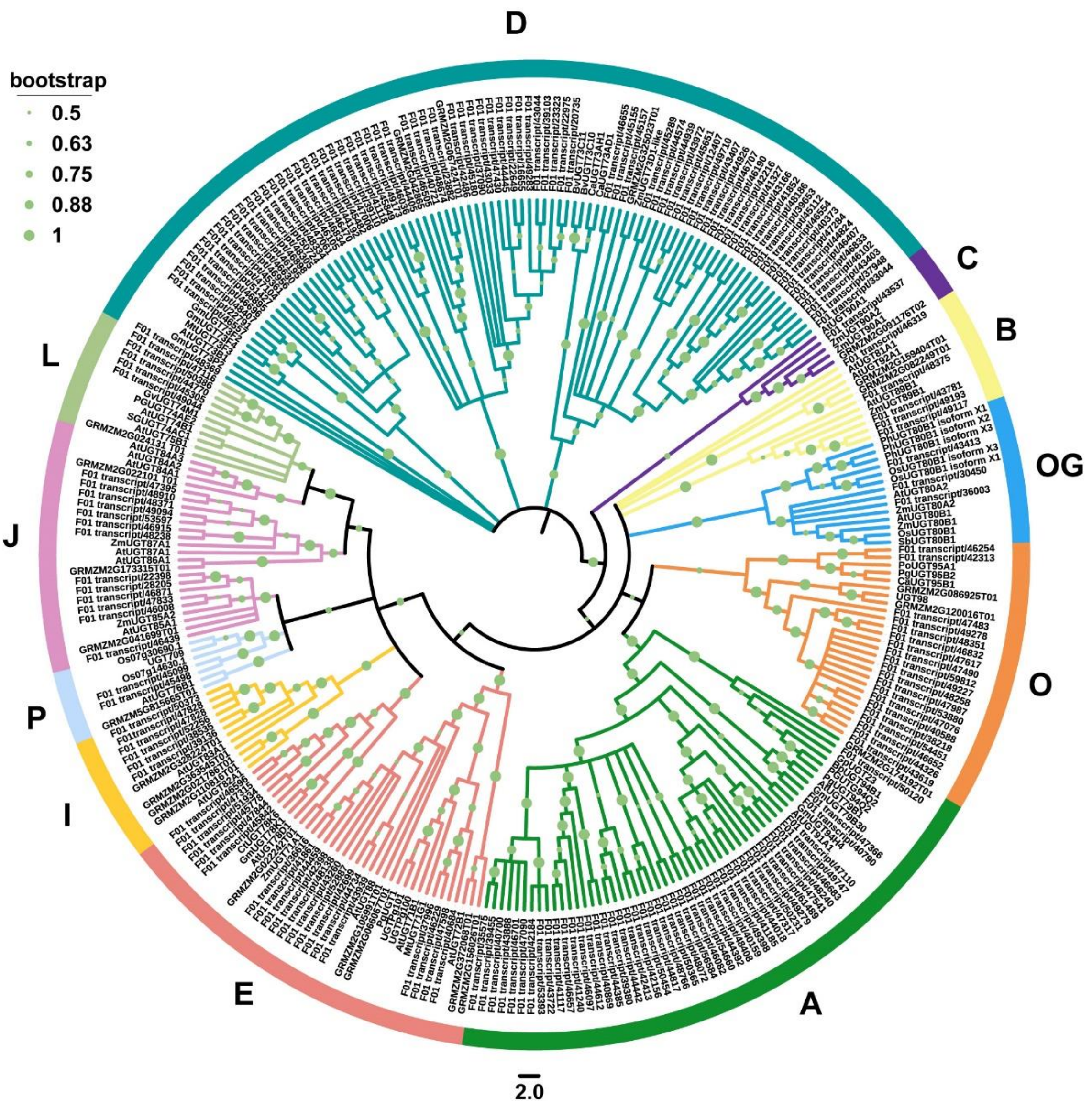

Supplementary Figure 2. Phylogenetic tree of UGTs. Predicted amino acid sequences of UGTs were aligned with selected UGTs from multiple species using MUSCLE. A neighbor-joining tree was constructed using MEGA6. At, *Arabidopsis thaliana*; Bp, *Bellis perennis*; Bv, *Barbarea vulgaris*; Ca, *Cicer arietinum*; Ct, Crystal structure; Gm, Glycine max; Gv, *Gypsophila vaccaria*; Mt, *Medicago truncatula*; Os, *Oryza sativa*; Pg, *Panax ginseng*; Ph, *Petunia maxim hybrid*; Po, *Pilosella officinarum*; Sg, *Siraitia grosvenorii*; Zm, *Zea mays*; GRMZM, UGT sequences from maize sequence database.

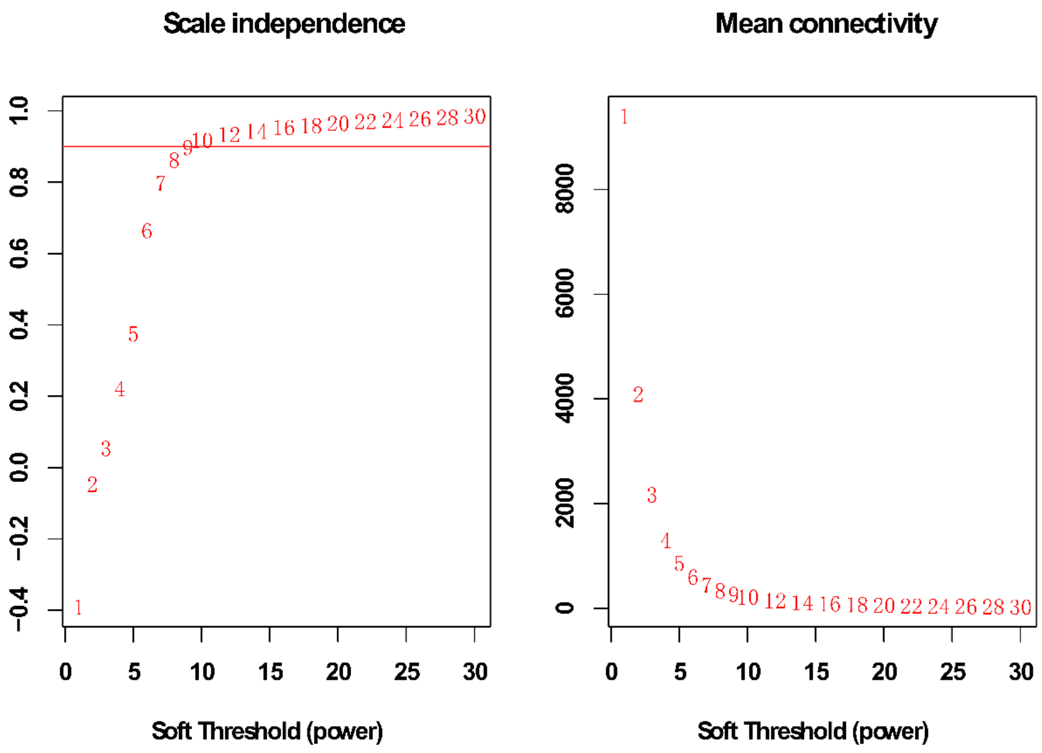

Supplementary Figure 3. dendrogram of 8 tissue samples from *Paris polyphylla* var. *yunnanensis*. The sample clustering tree is constructed by calculating the correlation coefficient of the expression level of each sample, and outlier samples are checked and removed.

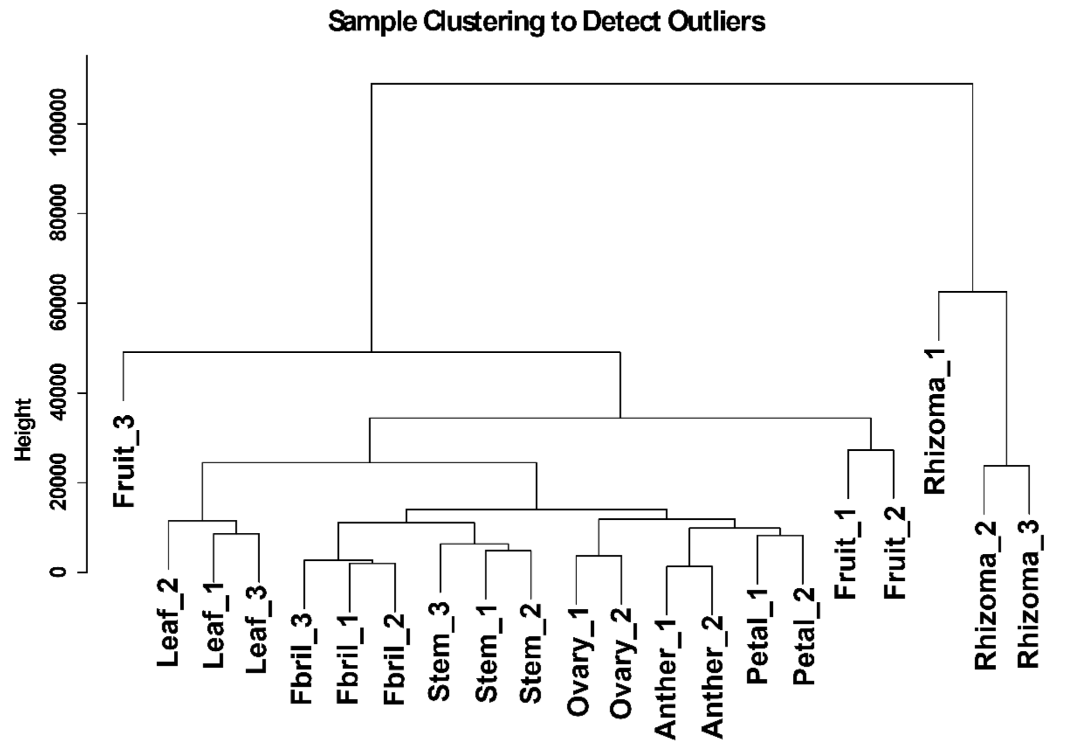

Supplementary Figure 4. Soft threshold determination of *Paris polyphylla* var. *yunnanensis* genes co-expression network (A) The scale-free fit index (y-axis) as a function of the soft-thresholding power (x-axis) and (B) the mean connectivity (degree, y-axis) as a function of the soft-thresholding power (x-axis).

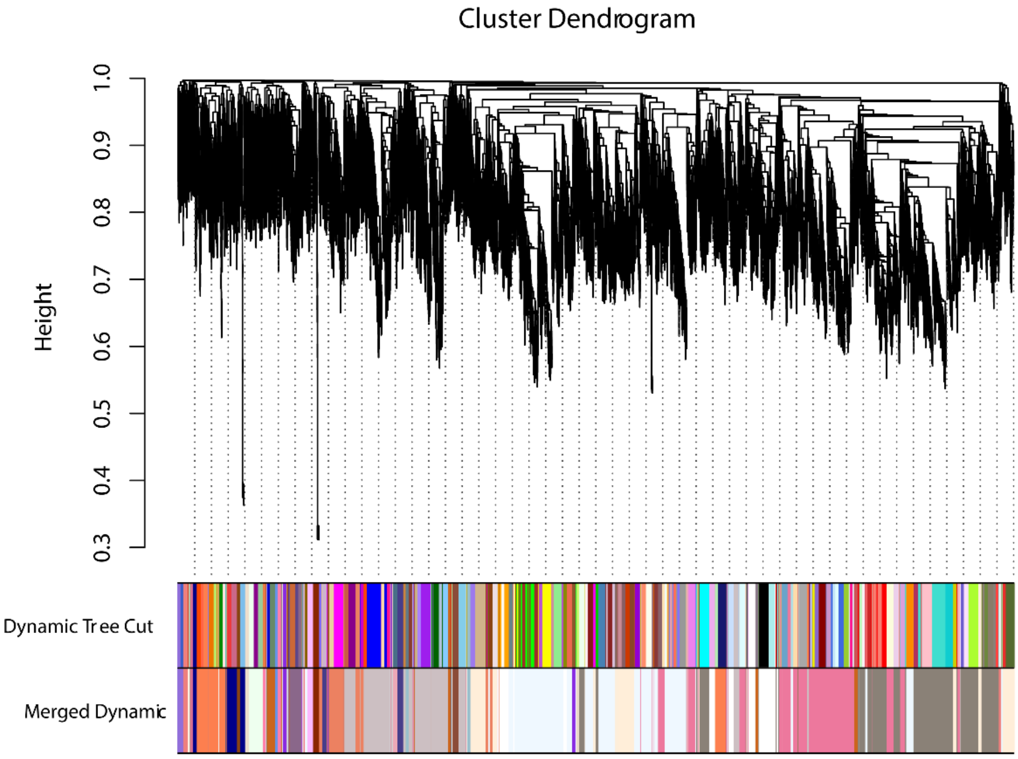

Supplementary Figure 5. Clustering dendrograms of *Paris polyphylla* var. *yunnanensis* genes. Each clade corresponds to a module and is marked with a different color.

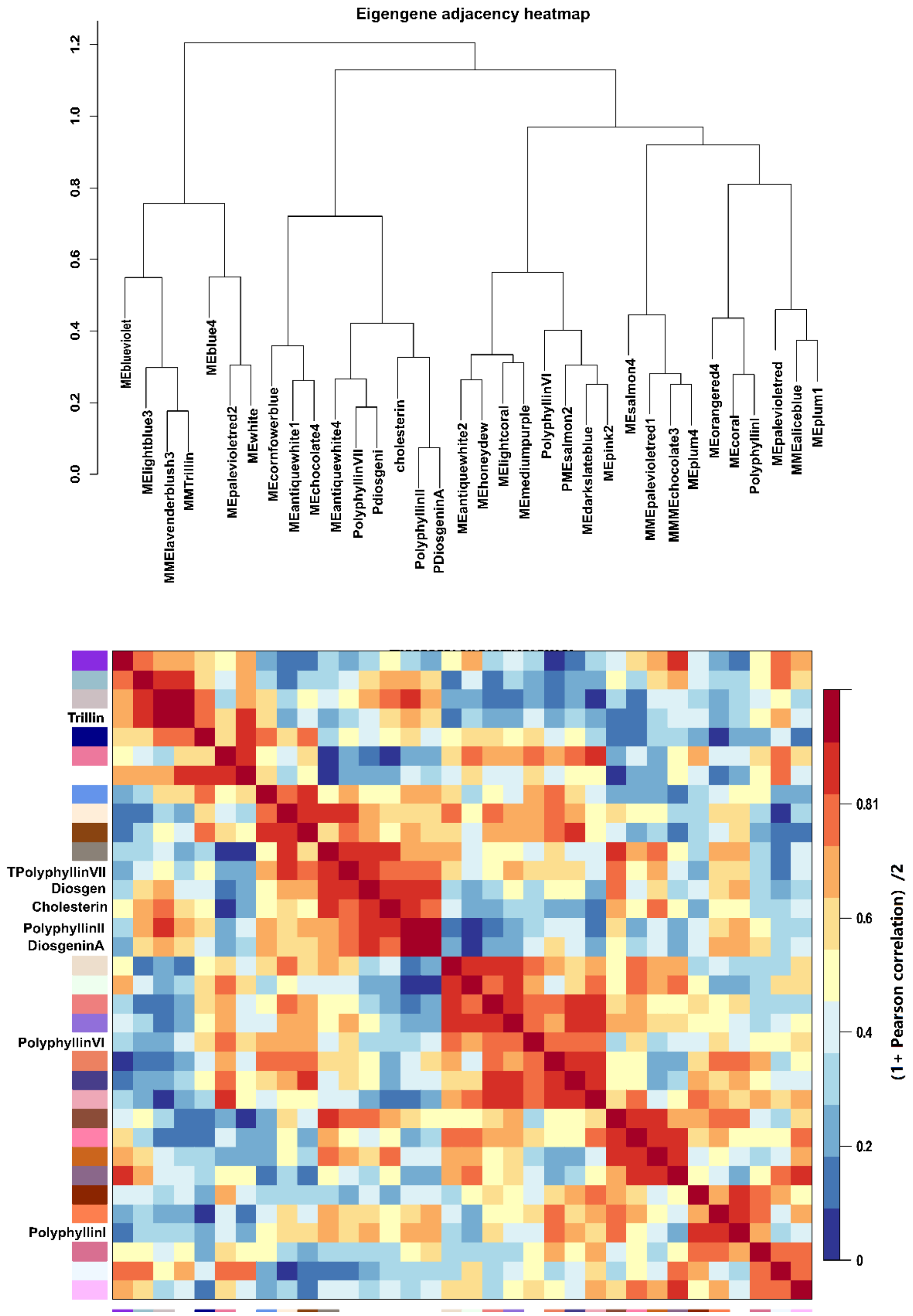

Supplementary Figure 6. Visualizing genes and metabolites co-expression network using a heatmap plot. The heatmap depicts the topological overlap matrix among all genes with the key metabolites in the biosynthetic pathway of polyphyllin. The lighter color represents the low correlation between the genes and metabolites biosynthesis,

and the gradually darker color represents the strong correlation between the genes and metabolites biosynthesis.

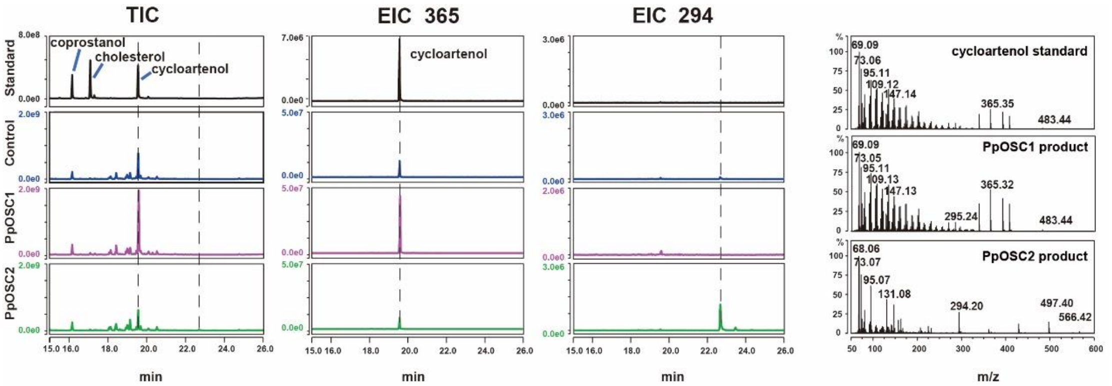

Supplementary Figure 7. Functional verification of *PpOSC* gene. Two OSC genes were identified in *P. polyphylla* var. *yunnanensis*, and the results of GC-MS showed that *PpOSC1* gene increased the yield of cycloartenol after being transferred to *N.benthamiana*.

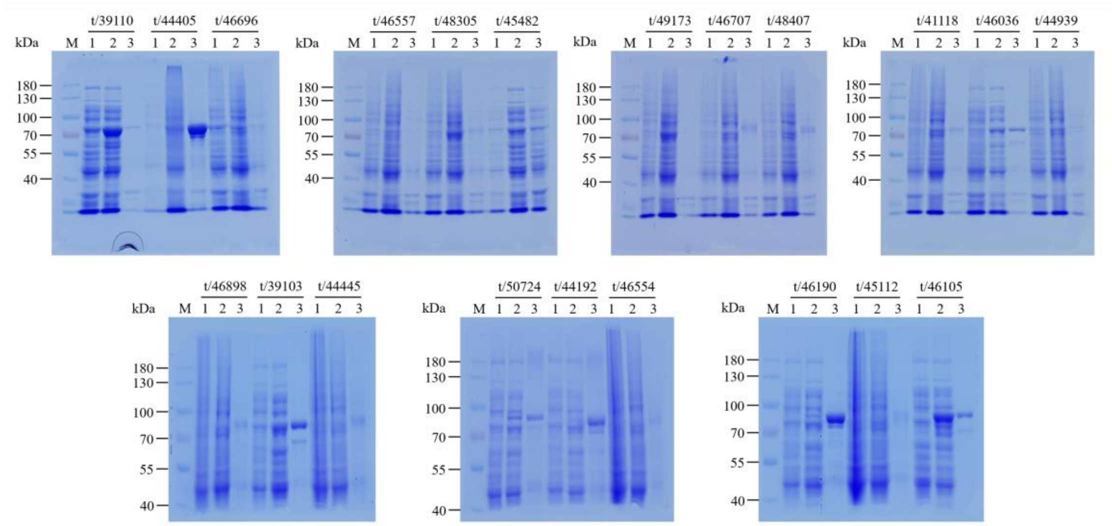

Supplementary Figure 8. SDS-PAGE analysis of the remaining candidate PpUGTs. *Lanes*: M, protein molecular weight marker (Thermo fisher); 1, pGEX-*PpUGT* vectors transformed in *E. coli* Rosetta (DE3) cells without IPTG induction; 2, supernatant of pGEX-*PpUGT* vectors transformed in *E. coli* Rosetta (DE3) cells with IPTG induction; 3, pellet of pGEX-*PpUGT* vectors transformed in *E. coli* Rosetta (DE3) cells with IPTG induction.

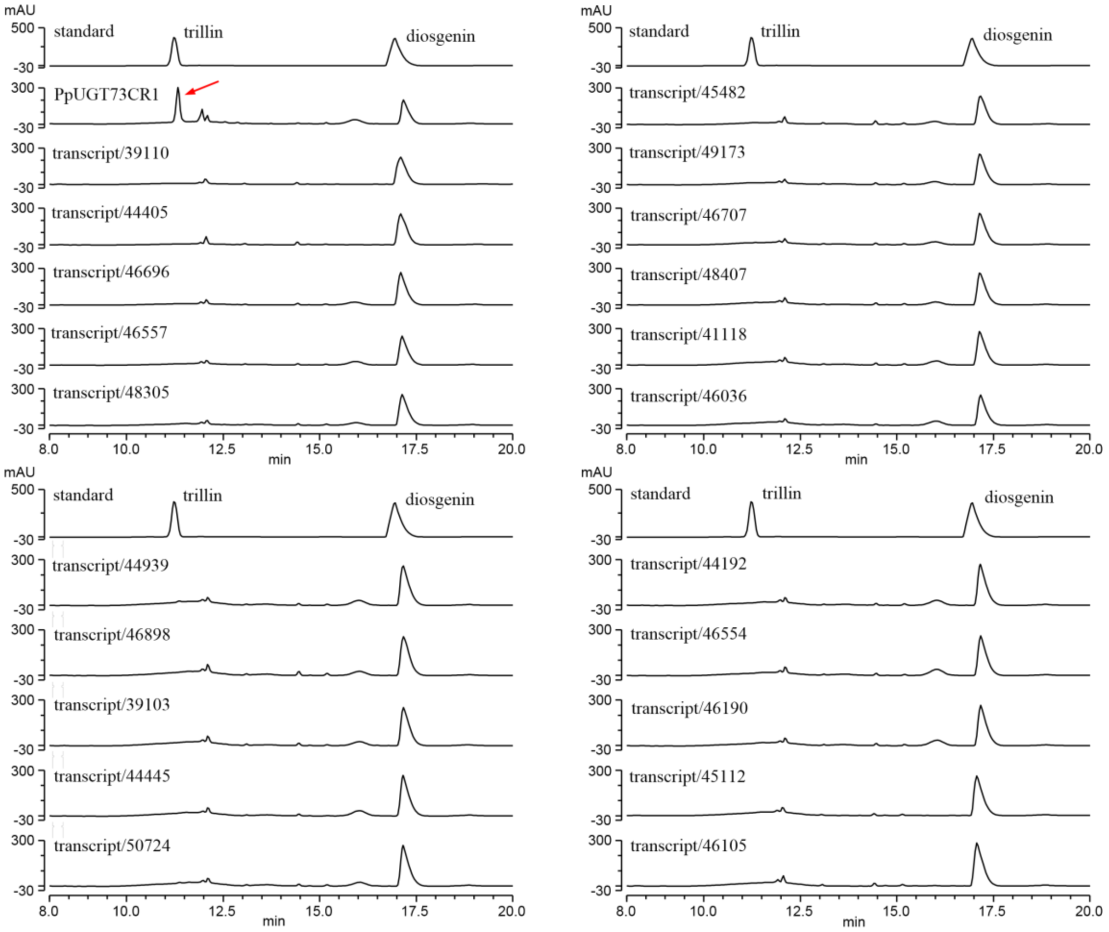

Supplementary Figure 9. HPLC analysis of diosgenin glycosylation catalyzed by candidate PpUGTs

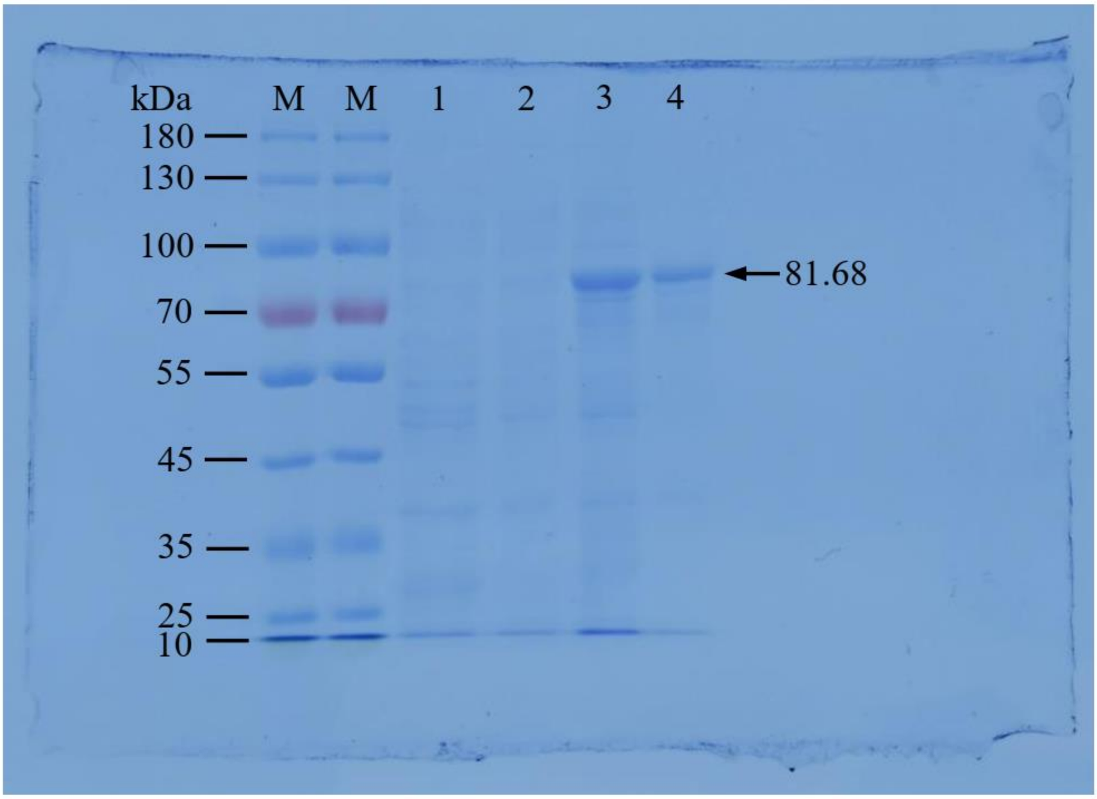

Supplementary Figure 10. SDS-PAGE analysis of expressed PpUGT73CR1 protein.

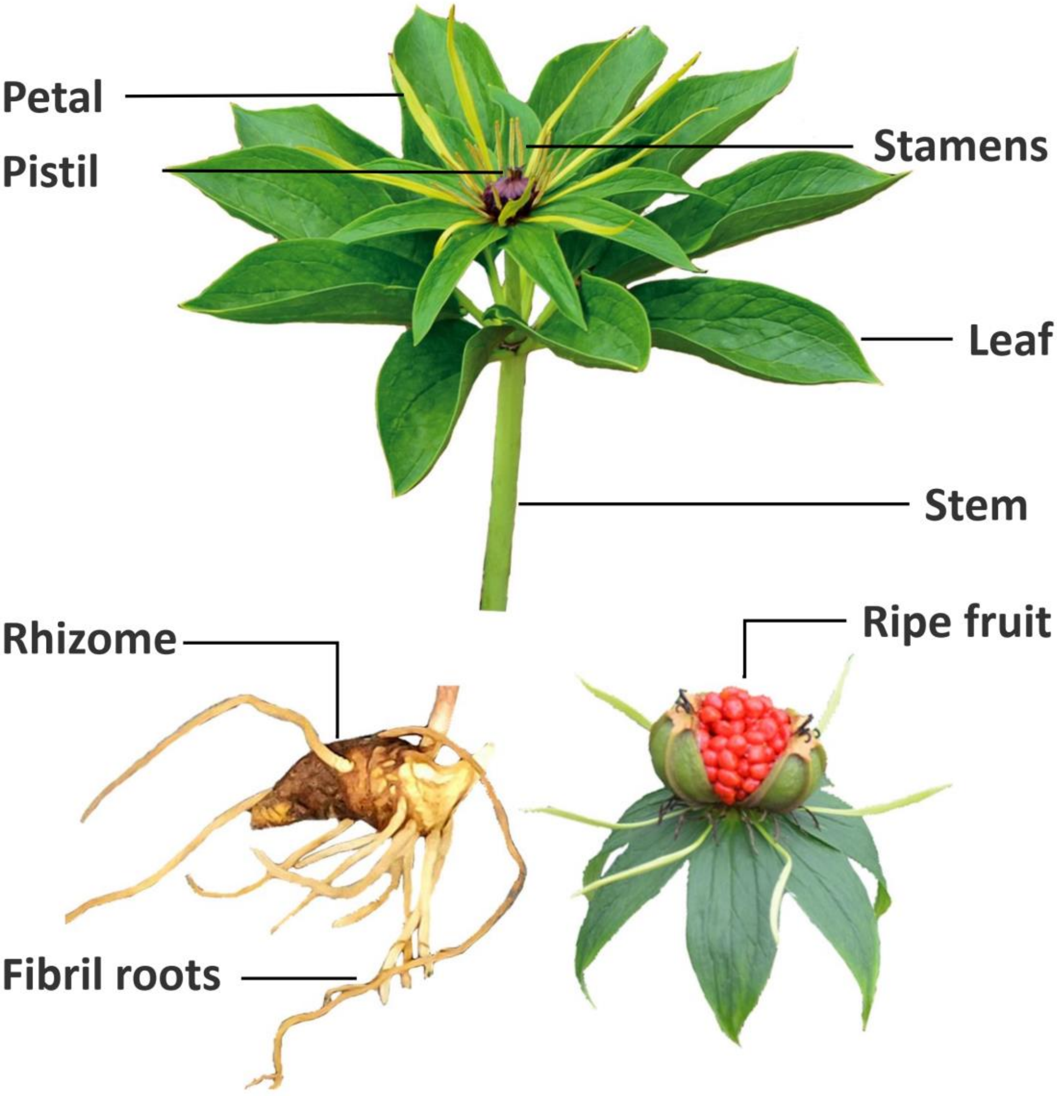

Supplementary Figure 11. Different organs of *P. polyphylla* var. *yunnanensis* used for transcriptomic and metabolomic analysis.

Supplementary Tables

Supplementary Table 1. Statistical table of transcriptome clean data of tissues in *Paris polyphylla* var. *yunnanensis*.

Samples	Read Number	Base Number	GC Content	≥Q30
Fibr1YD	49,181,509	14,677,368,556	51.43%	94.36%
Fibr2YD	48,722,996	14,530,314,100	50.98%	93.85%
Fibr3YD	47,968,347	14,279,858,192	51.23%	94.41%
Frui1YD	41,058,810	12,253,675,238	50.91%	94.51%
Frui2YD	41,408,373	12,360,642,172	50.45%	94.35%
Frui3YD	41,559,959	12,402,733,534	50.84%	94.12%
Leaf1YD	41,759,276	12,462,943,898	50.98%	94.51%
Leaf2YD	41,293,042	12,318,843,690	50.90%	94.42%
Leaf3YD	49,031,482	14,628,544,714	50.74%	94.65%
Rhiz1YD	40,972,867	12,240,818,562	51.59%	94.10%
Rhiz2YD	41,581,302	12,411,327,902	51.39%	94.60%
Rhiz3YD	41,955,499	12,528,751,962	51.80%	94.63%
Stem1YD	41,926,855	12,492,177,446	50.19%	94.08%
Stem2YD	40,316,576	12,039,632,420	50.13%	94.03%
Stem3YD	43,876,436	13,108,713,088	50.95%	94.16%
AB-2	41,208,394	12,316,381,062	51.71%	94.15%
AB-3	62,353,157	18,617,620,552	52.02%	94.43%
AY-2	58,423,460	17,456,235,974	52.19%	94.27%
AY-3	61,158,841	18,269,204,240	52.37%	94.27%
AZF-2	56,419,925	16,873,584,024	52.47%	94.15%
AZF-3	48,235,606	14,415,014,318	51.52%	94.38%

Read Number：Total number of pair-end Reads in Clean Data；Base Number：The total number of bases in Clean Data；GC Content：The percentage of G and C bases of total bases in Clean Data；≥Q30：The percentage of bases with a quality value of ≥ 30

Supplementary Table 2. The number of genes involved in polyphyllin biosynthesis pathway in WGCAN module.

**Modules**	**AACT**	**HMGS**	**HMGR**	**MVK**	**PMK**	**MPDC**	**Ipl**	**GPPS**	**FPS**	**TPS**	**SQS**	**SQE**	**CAS**	**SSR2**	**SMO3**	**CPI**	**CYP51**	**C14-R**	**8,7 SI**	**SMO4**	**C5-SD2**	**7-DR2**	**Totle**
**aliceblue**	0	1	1	0	1	0	1	0	0	3	3	0	2	1	2	0	0	1	2	1	1	0	**20**
**palevioletred2**	0	4	0	0	0	1	1	0	0	5	1	1	2	0	1	0	0	0	0	1	0	0	**17**
**coral**	0	1	1	0	0	0	0	0	0	0	0	2	0	7	1	0	0	1	0	1	0	0	**14**
**antiquewhite4**	2	3	3	1	0	1	0	0	0	2	0	2	1	3	2	0	0	0	0	1	0	0	**21**
**lavenderblush3**	0	1	1	0	0	0	0	0	2	4	1	0	0	0	0	0	0	0	0	0	0	2	**11**
**antiquewhite1**	0	0	0	0	0	1	0	0	0	1	0	0	0	1	1	0	0	0	1	0	0	1	**6**
**white**	0	0	0	1	0	0	0	0	0	1	0	1	0	1	0	0	0	0	0	0	0	0	**4**
**salmon2**	0	1	0	0	0	0	0	0	0	1	1	0	0	1	0	0	0	0	0	2	0	0	**6**
**honeydew**	1	1	0	0	2	0	0	0	0	1	0	0	0	0	0	0	0	0	1	0	2	1	**9**
**lightcoral**	0	0	0	0	0	1	0	0	0	0	0	0	0	0	0	0	0	0	0	0	0	0	**1**
**blue4**	0	0	0	0	0	0	0	0	0	0	1	0	0	0	0	0	0	0	0	0	0	0	**1**
**darkslateblue**	0	0	0	0	0	0	0	0	1	0	0	0	0	0	0	0	0	0	0	0	0	0	**1**
**plum4**	0	0	0	0	0	0	0	0	0	0	0	0	0	0	0	0	0	0	0	0	0	0	**0**
**antiquewhite2**	0	0	0	0	0	0	0	0	0	2	1	0	0	0	0	0	0	0	1	0	0	0	**4**
**mediumpurple**	0	0	0	0	0	0	0	0	0	3	0	0	0	0	0	0	0	0	0	0	0	0	**3**
**lightblue3**	0	0	0	0	0	0	0	0	0	0	0	0	0	0	0	0	0	0	0	0	0	0	**0**
**chocolate3**	0	1	0	0	0	0	0	0	0	0	0	0	1	0	0	0	0	0	0	0	0	0	**2**
**palevioletred**	0	0	0	0	0	0	0	0	0	0	0	0	0	0	0	0	0	0	0	0	0	0	**0**
**plum1**	0	0	0	0	0	0	0	0	0	0	0	0	0	0	0	0	0	0	0	0	0	0	**0**
**cornflowerblue**	0	1	0	0	0	0	0	0	0	0	0	0	1	0	0	0	0	0	0	0	0	0	**2**
**pink2**	0	0	0	0	0	0	1	0	0	0	0	0	0	0	0	0	0	0	0	0	0	0	**1**
**orangered4**	0	0	0	0	0	0	0	0	0	0	0	0	0	0	0	0	0	0	0	0	0	0	**0**
**blueviolet**	0	0	0	0	0	0	0	0	0	0	0	0	0	0	0	0	0	0	0	0	0	0	**0**
**salmon4**	0	0	0	0	0	0	0	0	0	0	0	0	0	0	0	0	0	0	0	0	1	0	**1**

Supplementary Table 3. Experimental conditions of LC-MS-MS for determination of polyphyllin in *Paris polyphylla* var. *yunnanensis*.

	Parent Ion	Daughter Ion	Polarity	DP	EP	CE	CXP
Diosgenin	413	269.1	ESI-	-100	-10	-35	-32
Trillin	575.2	119	ESI-	-100	-10	-21	-32
Prosapogenin	721.4	575.3	ESI-	-100	-10	-10	-32
Polyphyllin I	853.5	721.3	ESI-	-100	-10	-40	-32
Polyphyllin II	1013.9	721.6	ESI-	-100	-10	-55	-32
Polyphyllin VI	867.8	721.4	ESI-	-100	-10	-30	-32
Polyphyllin VII	736.8	119.1	ESI-	-100	-10	-55	-32
Digitoxin	763.5	503.3	ESI-	-100	-10	-35	-32

Supplementary Table 4. Strains and plasmids used in functional verification of OSC gene.

Plasmids and strains	Description	Reference
pδBLE2.0	Yeast expression vector	Yuan et al.(2016)
pδHis	pδBLE2.0 derivative with HIS3 gene.	This study
pδHis-PpOSC1	pδHis derivative with *PpOSC1* gene.	This study
pδHis-PpOSC2	pδHis derivative with *PpOSC2* gene.	This study
OPC1	One isolated variant derived from CEN.PK2-1C with an orthogonal cytosolic FPP biosynthetic pathway under the control of galactose-inducible promoter: *ERG10*, *ERG13*, *HMG1/2*, *ERG12*, *ERG8*, *ERG19*, *IDI1* and *ERG20*	Yuan et al.(2016)
BY-SQ1	OPC1（gal80Δ::ERG9）	Yuan et al.
SQ-PpOSC1	One isolated variant derived from BY-SQ1 with a *PpOSC1* gene under the control of galactose-inducible promoter	This study
SQ-PpOSC2	One isolated variant derived from BY-SQ1 with a *PpOSC2* gene under the control of galactose-inducible promoter	This study

Jifeng Yuan and Chi-Bun Ching. Mitochondrial acetyl-CoA utilization pathway for terpenoid productions. Metabolic Engineering, 2016, 38, 303–309.

Supplementary Note 1

Pennogenin (1): white amorphous powder; 1H-NMR (500 MHz, CD3OD) δH 0.81 (3H, d, J = 6.0 Hz, H-27), 0.85 (3H, s, H-18), 0.90 (3H, d, J = 7.5 Hz, H-21), 1.05 (3H, s, H-19), 3.50 (1H, m, H-3), 4.02 (1H, dd, J = 7.5 Hz, 6.5 Hz, H-16), 5.36 (1H, m, H-6); 13C-NMR (125MHz, CD3OD) δC 38.5 (C-1), 33.2 (C-2), 72.4 (C-3), 43.0 (C-4), 142.3 (C-5), 122.2 (C-6), 32.3 (C-7), 32.9 (C-8), 51.4 (C-9), 37.8 (C-10), 21.7 (C-11), 32.5 (C-12), 45.5 (C-13), 53.9 (C-14), 31.3 (C-15), 90.6 (C-16), 91.3 (C-17), 17.49 (C-18), 19.9 (C-19), 45.8 (C-20), 9.1 (C-21), 110.9 (C-22), 32.1 (C-23), 29.4 (C-24), 33.3 (C-25), 67.7 (C-26), 17.52 (C-27).

Floribundasaponin A (2): white amorphous powder; 1H-NMR (800 MHz, CD3OD) δH 0.81 (3H, d, J = 6.4 Hz, H-27), 0.85 (3H, s, H-18), 0.90 (3H, d, J = 7.2 Hz, H-21), 1.06 (3H, s, H-19), 3.16 (1H, t, J = 8.0 Hz, H-2′), 3.27 (1H, m, H-3), 3.66 (1H, dd, J = 11.2 Hz, 5.6 Hz, H-6′a), 3.86 (1H, brd, J = 11.2 Hz, 5.6 Hz, H-6′b), 4.02 (1H, t, J = 6.4 Hz, H-16), 4.39 (1H, d, J = 8.0 Hz, H-1′), 5.40 (1H, m, H-6); 13C-NMR (200 MHz, CD3OD) δC 38.5 (C-1), 33.3 (C-2), 77.9 (C-3), 39.7 (C-4), 142.0 (C-5), 122.5 (C-6), 32.1 (C-7), 32.9 (C-8), 51.5 (C-9), 38.0 (C-10), 21.7 (C-11), 32.5 (C-12), 45.9 (C-13), 53.9 (C-14), 31.3 (C-15), 90.6 (C-16), 91.3 (C-17), 17.49 (C-18), 19.8 (C-19), 45.5 (C-20), 9.1 (C-21), 111.0 (C-22), 32.1 (C-23), 29.4 (C-24), 30.7 (C-25), 67.7 (C-26), 17.52 (C-27), 102.5 (C-1′), 75.1 (C-2′), 79.8 (C-3′), 71.7 (C-4′), 78.1 (C-5′), 62.8 (C-6′).
