# Supplementary material for: Effective prediction of biosynthetic pathway genes involved in bioactive polyphyllins in Paris polyphylla
Source: Commun Biol. 2022 Jan 13;5:50. doi: 10.1038/s42003-022-03000-z (PMC8758714; doi:10.1038/s42003-022-03000-z)
Supplement: Supplementary file 3 — Description of Additional Supplementary Files [file 42003_2022_3000_MOESM3_ESM.pdf]

Description of Additional Supplementary Files

File name: Supplementary Data 1
Description: Original data for quantification of Polyphyllins contents.

File name: Supplementary Data 2
Description: OSC gene information of other plants in the OSC phylogenetic tree.

File name: Supplementary Data 3
Description: Primer sequences for predicting UGT genes involved in the biosynthesis of polyphyllin.
